# Addressing the Challenges of Hepatitis C Virus Resistance and Treatment Failure

**DOI:** 10.3390/v8080226

**Published:** 2016-08-16

**Authors:** Che C. Colpitts, Thomas F. Baumert

**Affiliations:** 1 Institut de Recherche sur les Maladies Virales et Hépatiques, Inserm, U1110, 3 rue Koeberlé, 67000 Strasbourg, France; colpitts@unistra.fr; 2 Université de Strasbourg, 67000 Strasbourg, France; 3 Institut Hospitalo-Universitaire, Pôle Hépato-Digestif, Hopitaux Universitaires de Strasbourg, 67000 Strasbourg, France

**Keywords:** hepatitis C virus, antiviral therapy, antiviral resistance, treatment failure

## Abstract

Chronic hepatitis C is a major cause of chronic liver disease, including liver cirrhosis and hepatocellular carcinoma. The development of direct-acting antivirals (DAAs) revolutionized hepatitis C virus (HCV) treatment by offering genuine prospects for the first comprehensive cure of a chronic viral infection in humans. While antiviral resistance is a significant limitation for interferon-based therapies, resistance and treatment failure still appear to be present in a small fraction of patients even in state-of-the-art DAA combination therapies. Therefore, treatment failure and resistance still remain a clinical challenge for the management of patients not responding to DAAs. In this special issue of *Viruses* on HCV drug resistance, mechanisms of antiviral resistance for different classes of antiviral drugs are described. Furthermore, the detection and monitoring of resistance in clinical practice, the clinical impact of resistance in different patient groups and strategies to prevent and address resistance and treatment failure using complementary antiviral strategies are reviewed.

Chronic hepatitis C is a major global cause of liver disease, including liver cirrhosis and hepatocellular carcinoma. More than 130 million persons, approximately 3% of the global population, are chronically infected with the hepatitis C virus (HCV). In the USA, chronic hepatitis C infection, the most common cause of liver-related death and liver transplantation, recently surpassed human immunodeficiency virus (HIV) infection as a cause of mortality [1]. The development of direct-acting antivirals (DAAs) revolutionized HCV treatment by offering genuine prospects for the first comprehensive cure of a chronic viral infection in humans [2]. While antiviral resistance is a significant challenge for interferon-based therapies, drug resistance in state-of-the-art DAA combination therapies appears to be limited to a small fraction of patients. However, resistance does still arise, and distinct genotypes, patients with advanced liver disease, liver transplantation patients or patients with HIV-HCV coinfection may pose particular challenges [3]. In this special issue of *Viruses*, mechanisms of antiviral resistance for different classes of antiviral drugs are described. Furthermore, the detection and monitoring of resistance in clinical practice, the clinical impact of resistance in different patient groups and strategies to prevent and address resistance using complementary antiviral strategies are reviewed.

Resistant variants are seen in most patients who do not achieve a sustained virological response (SVR) [4], highlighting the fact that resistance is a critical determinant of treatment outcome. Mechanisms of resistance may vary depending on the class of DAA, the genotype of HCV and patient group, and so the understanding of these mutations has substantial clinical implications. Ahmed and Felmlee [5] describe the mechanisms of DAAs and resistance-associated mutations, and highlight clinically relevant resistance data to guide the choice and combination of DAAs used in therapy. Eltahla and colleagues [6] zoom in on DAAs that target the HCV RNA-dependent RNA polymerase (RdRp) and discuss the mode of action of different classes of RdRp inhibitors and mechanisms of antiviral resistance. The impact of these particular resistance-associated variants on treatment outcomes is reviewed. Perales and colleagues [7] describe the selection of DAA-resistant viruses through the lens of quasispecies dynamics, highlighting genetic and phenotypic barriers to resistance that could be used to diminish the probability of viral breakthrough during therapy.

Given that the outcome of DAA-based therapies may be affected by the selection of resistant variants, virological tools to monitor drug resistance both in the drug development stage as well as in the clinic are of critical importance. Fourati and Pawlotsky [8] describe diagnostic tools available to monitor HCV-resistance to new drugs in development, and also in patients receiving DAA therapy. Nonetheless, Chayama and Hayes [9] point out that the presence of resistance-associated variants (RAVs) does not necessarily preclude successful treatment, particularly considering that most RAVs are quickly lost in the absence of DAAs. They further highlight differences in treatment regimens between the USA and Japan and the potential implications for resistance challenges.

Despite the remarkable cure rates of DAAs, the pursuit of complementary antiviral strategies is still warranted for patient groups who do not respond to therapy, due to DAA resistance or other factors. For example, a substantial fraction (10%–20%) of patients with decompensated cirrhosis do not respond to current DAAs, and treatment is associated with adverse effects [10]. Furthermore, the ability of DAAs to prevent liver graft reinfection is still under clinical evaluation [11]. Roche and colleagues [12] provide an update on the impact of DAAs on HCV infection following liver transplant. Although great advances have been made in this area, viral resistance and treatment failure have been observed using state-of-the-art DAAs pre- or post-liver transplantation, which complicates the management of patients.

Zeisel and colleagues [13] describe host-targeting agents (HTAs) that target cellular factors involved in the HCV life cycle as a potential option to prevent and treat viral resistance, particularly in the context of liver transplantation. HTAs, some of which are in preclinical or clinical development, act via complementary mechanisms of action to DAAs and exhibit a higher genetic barrier to resistance. Furthermore, Lovelace and Polyak [14] point out that chronic viral infections cause an ongoing state of chronic inflammation, which may contribute to the development of HCV-induced liver disease. In this issue, they describe natural products as tools to investigate and modulate the cellular metabolic pathways that contribute to inflammation during chronic infection. This may open perspectives for novel strategies to combat virus-associated liver disease.

Finally, Mesalam and colleagues [15] describe in vivo HCV infection models that will be critical for the assessment of HCV therapy response and the emergence of resistant variants. In particular, the evolution in the past decade of animal models enabling preclinical assessment of drug efficacy and resistance is discussed, focusing on the chimeric mice and chimpanzee models that have been critical in the past for identifying HCV antivirals and drug resistance mechanisms.

The development of DAAs markedly improved the outlook for HCV patients and provides the first real opportunity for global cure of a chronic viral infection. The novel strategies to prevent and address the challenges of DAA resistance described in this special issue of *Viruses* will help to bring the goal of global HCV eradication yet closer to reality.

## References

[1] Ly K.N., Xing J., Klevens R.M., Jiles R.B., Ward J.W., Holmberg S.D. (2012). The increasing burden of mortality from viral hepatitis in the United States between 1999 and 2007. Ann. Intern. Med..

[2] Chung R.T., Baumert T.F. (2014). Curing chronic hepatitis C—The arc of a medical triumph. N. Engl. J. Med..

[3] Sarrazin C. (2016). The importance of resistance to direct antiviral drugs in HCV infection in clinical practice. J. Hepatol..

[4] Pawlotsky J.M. (2016). Hepatitis C Virus Resistance to Direct-Acting Antiviral Drugs in Interferon-Free Regimens. Gastroenterology.

[5] Ahmed A., Felmlee D.J. (2015). Mechanisms of Hepatitis C Viral Resistance to Direct Acting Antivirals. Viruses.

[6] Eltahla A.A., Luciani F., White P.A., Lloyd A.R., Bull R.A. (2015). Inhibitors of the Hepatitis C Virus Polymerase; Mode of Action and Resistance. Viruses.

[7] Perales C., Quer J., Gregori J., Esteban J.I., Domingo E. (2015). Resistance of Hepatitis C Virus to Inhibitors: Complexity and Clinical Implications. Viruses.

[8] Chayama K., Hayes C.N. (2015). HCV Drug Resistance Challenges in Japan: The Role of Pre-Existing Variants and Emerging Resistant Strains in Direct Acting Antiviral Therapy. Viruses.

[9] Fourati S., Pawlotsky J.-M. (2015). Virologic Tools for HCV Drug Resistance Testing. Viruses.

[10] Curry M.P., O’Leary J.G., Bzowej N., Muir A.J., Korenblat K.M., Fenkel J.M., Reddy K.R., Lawitz E., Flamm S.L., Schiano T. (2015). Sofosbuvir and Velpatasvir for HCV in Patients with Decompensated Cirrhosis. N. Engl. J. Med..

[11] Felmlee D.J., Coilly A., Chung R.T., Samuel D., Baumert T.F. (2016). New perspectives for preventing hepatitis C virus liver graft infection. Lancet Infect. Dis..

[12] Roche B., Coilly A., Roque-Afonso A.-M., Samuel D. (2015). Interferon-Free Hepatitis C Treatment before and after Liver Transplantation: The Role of HCV Drug Resistance. Viruses.

[13] Zeisel M.B., Crouchet E., Baumert T.F., Schuster C. (2015). Host-Targeting Agents to Prevent and Cure Hepatitis C Virus Infection. Viruses.

[14] Lovelace E.S., Polyak S.J. (2015). Natural Products as Tools for Defining How Cellular Metabolism Influences Cellular Immune and Inflammatory Function during Chronic Infection. Viruses.

[15] Mesalam A.A., Vercauteren K., Meuleman P. (2016). Mouse Systems to Model Hepatitis C Virus Treatment and Associated Resistance. Viruses.

